# Comparison of cytotoxicity and genotoxicity of 4-hydroxytamoxifen in combination with *Tualang* honey in MCF-7 and MCF-10A cells

**DOI:** 10.1186/1472-6882-14-106

**Published:** 2014-03-19

**Authors:** Nik Soriani Yaacob, Nur Faezah Ismail

**Affiliations:** 1 Department of Chemical Pathology, School of Medical Sciences, Universiti Sains Malaysia, Health Campus, 16150 Kubang Kerian, Kelantan, Malaysia

**Keywords:** *Tualang* honey, Tamoxifen, MCF-10A, MCF-7, Cytotoxicity, DNA damage, DNA repair enzymes

## Abstract

**Background:**

The Malaysian *Tualang* honey (TH) is not only cytotoxic to human breast cancer cell lines but it has recently been reported to promote the anticancer activity induced by tamoxifen in MCF-7 and MDA-MB-231 cells suggesting its potential as an adjuvant for the chemotherapeutic agent. However, tamoxifen produces adverse effects that could be due to its ability to induce cellular DNA damage. Therefore, the study is undertaken to determine the possible modulation of the activity of 4-hydroxytamoxifen (OHT), an active metabolite of tamoxifen, by TH in non-cancerous epithelial cell line, MCF-10A, in comparison with MCF-7 cells.

**Methods:**

MCF-7 and MCF-10A cells were treated with TH, OHT or the combination of both and cytotoxicity and antiproliferative activity were determined using LDH and MTT assays, respectively. The effect on cellular DNA integrity was analysed by comet assay and the expression of DNA repair enzymes was determined by Western blotting.

**Results:**

OHT exposure was cytotoxic to both cell lines whereas TH was cytotoxic to MCF-7 cells only. TH also significantly decreased the cytotoxic effect of OHT in MCF-10A but not in MCF-7 cells. TH induced proliferation of MCF10A cells but OHT caused growth inhibition that was abrogated by the concomitant treatment with TH. While TH enhanced the OHT-induced DNA damage in the cancer cells, it dampened the genotoxic effect of OHT in the non-cancerous cells. This was supported by the increased expression of DNA repair proteins, Ku70 and Ku80, in MCF-10A cells by TH.

**Conclusion:**

The findings indicate that TH could afford protection of non-cancerous cells from the toxic effects of tamoxifen by increasing the efficiency of DNA repair mechanism in these cells.

## Background

Chemotherapeutic agents effectively kill most of the cancer cells. However, cellular specificity of those drugs remains a major obstacle where side effects often develop as a result of the drugs’ action on the normal cells and tissues. Tamoxifen is commonly used to treat breast tumors that are classified as estrogen receptor positive (ER+) since the 1980s [1,2]. It blocks the estrogenic effects responsible for growth and proliferation of breast cancer cells. However in certain cases, breast cancer patients present with intrinsic resistance to tamoxifen treatment [3] and increasing the dose will only lead to the problem of undesirable effects to the patients as a result of the drug action on normal tissues and cells. These include endometrial cancer risk [4], risk of liver cancers [5] and effect on eyesight [6]. Chromosomal aberrations and DNA damage have also been reported in several cell types and animal organs [7-9]. Tamoxifen undergoes metabolic reactions involving the cytochrome P450 enzyme family and lack of this enzyme family was suggested to lead to tamoxifen resistance of the cancer cells [10].

Honey is a natural product containing a complex mixture of sugars, minerals, proteins, vitamins, organic acids, flavonoids, phenolic acids, enzymes and other phytochemicals. It contains antioxidant molecules such as flavonoids, phenolic acids, catalase, carotenoids, peroxidase and catalase [11]. Honey’s many potential health benefits have amounted great interest in its use as an alternative remedy for various ailments by the general public [12]. Research has shown that honey is capable of inhibiting the growth of bladder cancer cell lines and is effective against murine bladder cancer implantation [13]. More recently, a Nigerian jungle honey displayed antitumour activity in mice inoculated with Lewis Lung Carcinoma cells [14] and Manuka honey inhibited the proliferation of murine melanoma, colorectal carcinoma and breast cancer cells as well as tumour growth in a melanoma tumour model [15]. *Tualang* honey (TH) is a wild multifloral Malaysian honey produced by *Apis dorsata* (Asian giant bees) that build their hives high up in the *Tualang* tree (*Koompassia excelsa*). We have recently shown that TH could inhibit growth of breast cancer cells, MCF-7 and MDA-MB-231, by inducing apoptosis [16] and promoted the anticancer effects of tamoxifen on these cells [17]. However, TH was observed to be noncytotoxic to the noncancerous breast epithelial cells, MCF-10A [16]. The present study was therefore carried out to further understand the differential effects of TH on MCF-7 and MCF-10A cells and to determine its influence on the activity of the tamoxifen metabolite, 4-hydroxytamoxifen (OHT) in these cells.

## Methods

### Cell culture and treatment

MCF-10A cell line was purchased from the American Type Culture collection and maintained in complete growth medium consisting of 1:1 mixture of Dulbecco’s modified Eagle’s medium and Ham’s F12 medium supplemented with 5% fetal bovine serum, 20 ng/ml epidermal growth factor, 10 μg/ml insulin, 500 ng/ml hydrocortisone and 1 unit/ml penicillin/streptomycin. TH was supplied by the Federal Agricultural Marketing Authority Malaysia. This type of honey is produced by Asian giant bees (*Apis dorsata*) that built their hives on *Tualang* trees (*Koompassia excels*) in the Malaysian jungle. Prior to cell treatment, the TH was freshly prepared by dissolving it in serum-free culture medium at a final concentration of 10% (v/v) and then was filter-sterilized using 0.22 μm syringe filter unit (Millipore, USA). The tamoxifen metabolite, OHT, was obtained from Sigma-Aldrich®, dissolved in ethanol and stored at −20°C in aliquots. Cells were treated with OHT, TH or their combination for up to 72 hr.

### Cytotoxicity assay

Cells were seeded in 24-well cell culture plates (Nunc, Denmark) at a density of 1 × 10^5^ cells/ml in a complete growth medium for 24 hr at 37°C in a humidified incubator. The culture medium was replaced with assay medium (2% fetal bovine serum and without epidermal growth factor) prior to treatment with TH (1%), OHT (10 or 15 μM) or their combination for up to 72 hr. Culture cell supernatants (100 μl) were transferred into a 96-well microplate and the cytotoxicity was assessed using Lactate Dehydrogenase (LDH) Cytotoxicity Detection Kit (Roche, Germany) according to the manufacturer’s instructions.

### Proliferation assay

Cells were seeded in a 96 well plate at a density of 2 × 10^4^ cells per well and treated with 1% TH, 10 μM OHT, 15 μM OHT and their combination for 24 and 48 hr. At the end of treatment, 20 μl of MTT (5 mg/ml) was added to each well and the plate was incubated for 4 h at 37°C with 5% CO_2_. The medium was then aspirated and 100 μl of DMSO was added to each well to dissolve the tetrazolium crystals produced by viable cells. Absorbance was measured using a plate reader (VersaMax, Molecular Devices, US) at 540 nm wavelength.

### Comet assay

Cells were seeded as above and treated with TH (1%), OHT (10 μM) and their combination of both for 24 hr. Cells were then collected by trypsinisation and washed with PBS. Comet assay analysis was performed by using Trevigen’s Commet Assay® kit (Trevigen, Inc) according to the manufacturer’s instructions. Briefly, cell pellets were mixed with low melting point agarose at a ratio of 1:10 (v/v) and pipetted onto the CometSlide™. Cells were then lysed in the lysis solution before been treated with alkali solution in order to unwind and denature the DNA and hydrolyze sites of damage. The samples were then electrophoresed, stained with a fluorescent DNA intercalating dye (SYBR® green 1) and visualized under fluorescence microscope (Nikon, Tokyo, Japan) using a band pass FITC filter (excitation 490 nm, emission >520 nm). Fifty cells were randomly selected and captured per sample using 100× magnification and were analyzed by using Comet Image Analysis System software (CometScores software; TriTek, Sumerduck, VA, USA) that is available at http://www.autocomet.com/products_cometscore.php. Tail length, % of DNA in the tail and Olive moment were used as evaluation of the DNA damage.

### Western blotting

Cells cultured in 75 cm^2^ flasks at a density of 1 × 10^6^ cells/ml were treated with TH (1%), OHT (10 μM) or their combination for 24 hr. Cell were lysed in 150 μl lysis buffer (50 mM of Tris–HCl, 150 mM of NaCl, 0.2% SDS, 1 mM PMSF, 2 μg/ml of leupeptin, 2 μg/ml of aprotinin and 1 mM of Na_3_VO_4_) and the lysates were obtained by centrifugation at 12,000 rpm for 2 min at 4°C. The protein concentrations were determined using a spectrophotometer (NanoDrop) at 280 nm absorbance. For western blot analysis, 100 μg of protein was resolved on 10% SDS-polyacrylamide gel for 1 hr followed by semi-dry transfer onto PVDF membrane and blocking using 5% skimmed milk diluted in the TBS-Tween 20. Next, the membrane was washed with TBS-Tween20 and incubated with the primary antibody (Rad 51, Ku70/Ku80 and β-actin; Abcam, USA) overnight at 4°C. After washing with TBS–Tween 20, the membrane was incubated with secondary antibody conjugated with horseradish peroxidase for 1 hr at room temperature. Antibody binding was detected by incubating the membrane with ECL™ Prime Western Blotting reagent according to the manufacturer’s protocols and visualized using an image analyzer under chemiluminescence filter. The band density for each treatment compared to control was analyzed using ImageJ 1.46 software (http://imagej.nih.gov/ij/) and the values were normalized to the β-actin band density.

### Statistical analysis

Triplicates of three independent experiments were carried out and data are expressed as the mean ± SD. Differences between the groups were evaluated by using Student T-test or one-way analysis of variance (ANOVA) followed by post hoc Tukey multiple comparison test with the aid of GraphPad Prism 5 software. p < 0.05 was taken as being statistically significant.

## Results and discussion

Various studies have recently been conducted to explore the medicinal benefits of Tualang honey and findings have shown that TH has significant anticancer activity against human cancer cell lines such as breast, cervical [16], oral and osteosarcoma [18]. Cancer cell death by TH occurs via induction of caspase-dependent apoptosis [16]. Recently, 7,12-dimethylbenz [a] anthracene (DMBA)-induced breast tumours in TH-treated rats were reported to be much less in number, volume and weight with better histological grade and morphology compared to the non-honey treated rats [19]. The anticancer activities showed by TH could be attributed to its antioxidant property as shown by the high total phenolic content and total antioxidant activity based on the ferric reducing ability of plasma (FRAP) assay [20]. Other than phenolic compounds, peptides, organic acids, enzymes and other minor components could also contribute to the antioxidant capacity of honey [11].

Tamoxifen is widely used as an antioestrogen for treatment of breast cancer [21] but several side effects have been noted including increased endometrial and colorectal cancer risk reviewed in [22] that could be attributed to the drug’s ability to form DNA adducts through α-hydroxylation of the parent molecule and its metabolites including OHT [23,24]. The drawbacks of chemotherapeutic agents have led to the search and development of new antitumor candidates or adjuvants. However in many cases non-cancerous cells often are not tested alongside the cancerous cells. The idea that TH activities could possibility be selective against cancerous cells came from an initial study which showed that human breast carcinoma cell lines, MCF-7 and MDA-MB-231, were highly sensitive to TH while the non-cancerous MCF-10A breast epithelial cells were not affected [16]. The present study was therefore carried out to further understand the differential effects of TH on MCF-7 and MCF-10A cells and to determine its influence on the activity of OHT in these cells. The dose of honey selected was based on our previously published concentration that caused apoptosis as well as promotion of tamoxifen-induced apoptosis of MCF-7 cells [17]. The concentration of OHT chosen was within the ranges reported in the literature for breast cancer cells including MCF-7 [25,26].

**Figure 1:**
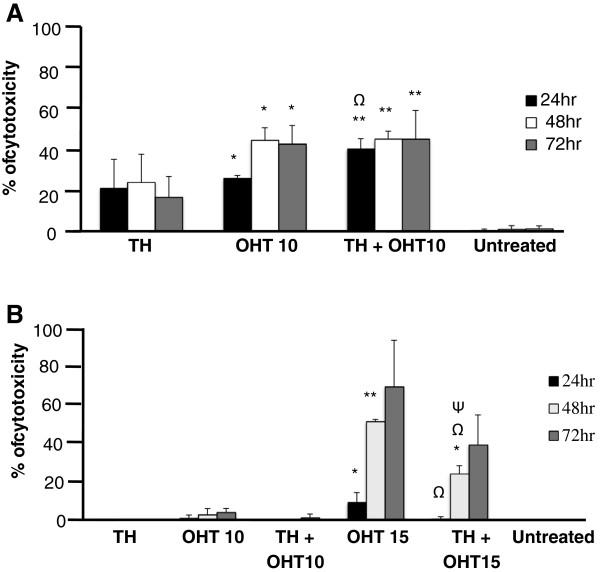
**Cytotoxicity of TH and OHT on MCF**-**7 and MCF**-**10A cells.** MCF-7 **(A)** and MCF-10A **(B)** cells were treated with OHT (10 and 15 μM), TH (1% v/v) and their combination for up to 72 hr and cell death was determined using LDH assay. Each value represents the mean ± SD from three independent experiments. Statistical significance was determined by using the Student T test. ^Ψ^p < 0.05 for TH + OHT compared to TH; ^Ω^p < 0.01 for TH + OHT compared to OHT; *p < 0.05, **p < 0.01 compared to untreated cells.

**Figure 2:**
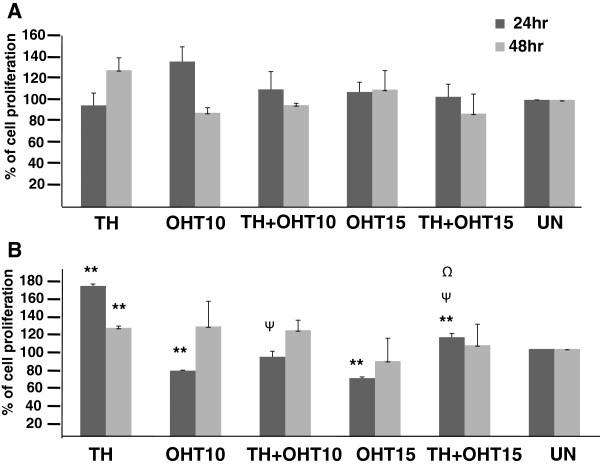
**Proliferation of MCF**-**7 and MCF**-**10A cells induced by TH and OHT.** MCF-7 **(A)** and MCF-10A **(B)** cells were treated with OHT (10 and 15 μM), TH (1%) and their combination for up to 48 hr followed by an MTT assay. Each value represents the mean ± SD that has been normalized against the respective control (taken as 100%). Statistical significance was determined by using the Student T test. ^Ψ^p < 0.01 for TH + OHT compared to TH; ^Ψ^p < 0.01 for TH + OHT compared to OHT; *p < 0.05, **p < 0.01 compared to untreated cells.

Both TH (1%) and OHT (10 μM) were found to be cytotoxic to MCF-7 cells with the latter being more effective with longer treatment duration (Figure 1A). In line with our previous findings of increased apoptosis with the combination of TH and tamoxifen [17], the current study also shows that TH promoted the cytotoxic effect of the tamoxifen derivative, OHT, in the cancerous MCF-7 cells from 25.2% to 38.9% within 24 hr. At 15 μM concentration, OHT was also cytotoxic to the non-cancerous MCF-10A cells and the effect increased with exposure time causing 70.1% cell death after 72 hr (Figure 1B). This is in agreement with the report of Petinari et al. [27] where tamoxifen was found to be cytotoxic to both cancerous and non-cancerous cell lines at micromolar concentrations and was in fact reported to be more aggressive in the non-cancerous cells. This was thought to be due to the higher expression of estrogen receptor by these non-cancerous cells. Interestingly, rather than promoting OHT’s activity as observed with the MCF-7 cells, TH significantly ameliorated the cytotoxic effect of the antioestrogen in MCF-10A cells at all time points (Figure 1B) suggesting TH’s protective effect in non-cancerous cells. The cytotoxicity results observed are supported by the findings of the MTT assay (Figure 2). TH was found to markedly increase the proliferation of MCF10A cells while not significantly affecting the proliferation of MCF-7 cells. OHT on the other hand, reduced the proliferation of MCF-10A cells but the presence of TH partially abrogated this inhibition. Proliferation of MCF-7 cells was not significantly modulated by all treatments. Tamoxifen induces chromosomal breaks [7] and is genotoxic to both normal lymphocytes and breast cancer cells [9]. Formation of DNA single- and double-strand breaks and oxidative modifications of purines and pyrimidines in these cells were thought to involve free radical generation [9].

**Figure 3:**
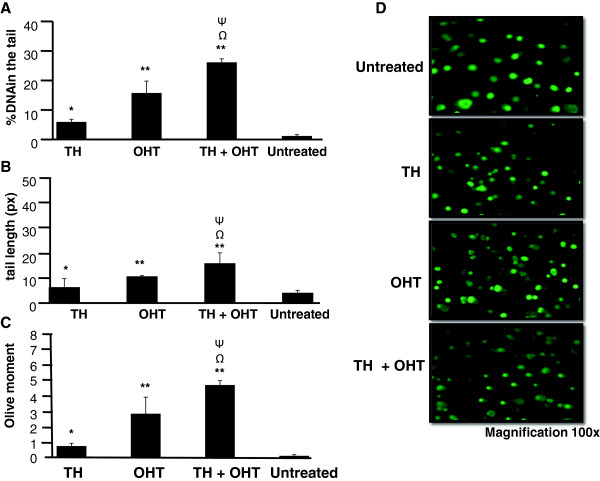
**DNA damage induced by TH and OHT in MCF**-**7 cells.** Comet assay was performed following treatment of cells with OHT (10 μM), TH (1%) and their combination for 24 hr. Cells were stained with SYBR Green and analyzed using Comet Image Analysis System software. Percentage of DNA in the tail **(A)**, tail length **(B)** and Olive moment **(C)** were used as evaluation of DNA damage. The values are presented as means ± SD from three independent experiments. Cells were observed under fluorescence microscopy **(D)**. Statistical analysis was determined using one-way ANOVA followed by post hoc Tukey multiple comparison test. ^Ψ^p < 0.05 for TH + OHT compared to TH; ^Ω^p < 0.01 for TH + OHT compared to OHT; *p < 0.05, **p < 0.01 compared to untreated cells.

**Figure 4:**
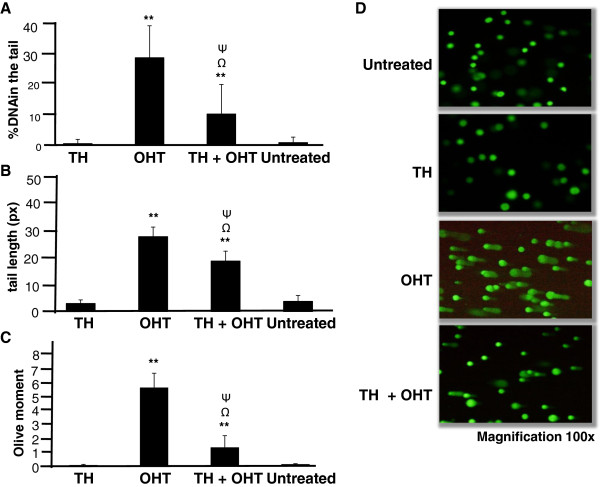
**DNA damage induced by TH and OHT in MCF**-**10A cells.** Comet assay was performed following treatment of cells with OHT (10 μM), TH (1%) and their combination for 24 hr. The cells were stained with SYBR Green and analyzed using Comet Image Analysis System software. Percentage of DNA in the tail **(A)**, tail length **(B)** and Olive moment **(C)** were used as evaluation of DNA damage. The values are presented as means ± SD from three independent experiments. Cells were observed under fluorescence microscopy **(D)**. Statistical analysis was determined using one-way ANOVA followed by post hoc Tukey multiple comparison test. ^Ψ^p < 0.05 for TH + OHT compared to TH; ^Ω^p < 0.01 for TH + OHT compared to OHT; **p < 0.01 compared to untreated cells.

In the current study, comet assay was performed to evaluate total cellular DNA damage. A suboptimal concentration (10 μM) of OHT was used for the comet assay so that at least the required 90% living cells were present to avoid false positive results (according to the manufacturer’s recommendations). Assessment of percentage of DNA in the comet tail, the tail length and Olive moment show that OHT induced significant DNA damage in MCF-7 cells (Figure 3) as well as MCF-10A cells (Figure 4), indicating that accumulation of DNA damage is involved in OHT-induced cytotoxicity in both cancerous and non-cancerous cell lines. OHT was found to be more genotoxic to MCF-10A than MCF-7 cells with DNA content in the tail of 27.2% and 15.8%, respectively. MCF-10A cells also displayed higher values of tail length and Olive moment. On the other hand, TH induced DNA damage only in MCF-7 cells and did not affect the integrity of DNA in MCF-10A cells.

We then show that the combination of OHT and TH promoted more extensive DNA damage in the cancerous MCF-7 cells as reflected by the significantly higher percentage of DNA in the tail of the comet (increased from 15.8 to 26.1%), as well as the increased tail length and olive moment compared to OHT treatment alone (Figure 3). In contrast, TH significantly reduced the DNA damage induced by OHT (reduced from 27.2 to 9.6%) in MCF-10A cells (Figure 4). Wozniak et al. [9] reported that DNA strand breaks induced by tamoxifen were more persistent in normal cells compared to cancerous cells probably because of less effective repair mechanisms. Long-term exposure to this drug may therefore induce mutations in critical genes especially those involved in the DNA repair pathways. Repair of DNA lesions is therefore critical for the cell to preserve the integrity of its genomic and incomplete repair could later lead to malignant transformation [28,29]. According to Ellsworth et al. [30], genetic abnormalities have been observed in histologically normal epithelial cells surrounding breast carcinomas and that abnormalities of tumourigenic importance could accumulate in these cells. It could therefore be inferred from the current findings that TH may have the ability to repair and/or preserve genomic stability in the normal cells.

We further examined whether differences in DNA damage effects in the cancerous and non-cancerous cells induced by TH is associated with modulation of the expression of Rad51, Ku70 and Ku80 enzymes which are involved in the repair of DNA double strand breaks. Rad51 plays a role in the strand break repair through homologous recombination while Ku70 and Ku80 are involved in non-homologous end joining repair pathway without the requirement for a homologous template [31]. Although they both form a heterodimer, it is possible that they function independently [32]. Inability to repair DNA damage could lead to genetic instability or mutation or chromosomal aberration that could enhance cancer development [31]. Analysis of more than 100 tumour specimens revealed that Rad51 over expression significantly correlates with mammary tumour grading and is considered as a potential biomarker for diagnosis and prognosis of invasive ductal mammary carcinoma [33]. In a study conducted by Pucci et al. [34], Ku70 and Ku80 heterodimer binding activity and the heterodimer DNA repair capability were reduced in advanced breast and bladder tumours [34].

**Figure 5:**
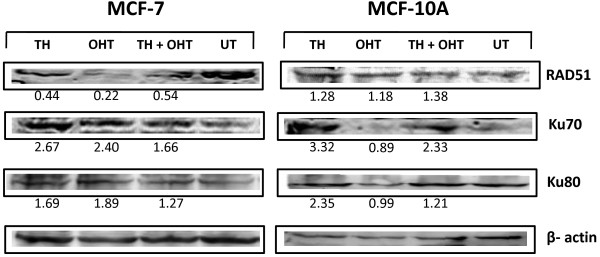
**Expression of DNA repair enzymes in MCF**-**7 and MCF**-**10A cells treated with TH and OHT.** The expression of Rad51, Ku70 and Ku80 in the cells was determined by Western blotting following 24 hr exposure to OHT (10 μM), TH (1%) or their combination. The protein bands were visualized using an image analyzer and band density was analysed using ImageJ software with fold-change difference of expression compared to untreated cells (UT) written below each band.

Our results show that Rad51 protein was not upregulated by either OHT or TH alone or their combination indicating a non-significant role of this repair enzyme. In contrast, both OHT and TH increased Ku70 (2.40- and 2.67-fold, respectively) and Ku80 (1.89- and 1.69-fold, respectively) expression in MCF-7 cells. However in MCF-10A cells, a marked overexpression of Ku70 (3.32-fold) and Ku80 (2.35-fold) was observed following TH treatment but not by OHT (Figure 5). The presence of TH together with OHT in MCF-10A cells upregulated the expression of Ku70 but not Ku80 in comparison with OHT treatment alone. The results suggest for the ability of TH to enhance the capability of DNA double strand DNA repair through the non-homologous end-joining repair pathway, especially in the non-cancerous breast epithelial cells. We saw earlier that TH dampened the DNA damage induced by OHT in the noncancerous cells and this is corroborated by the overexpression of Ku70 DNA repair enzyme in cells treated with both TH and OHT compared to OHT treatment alone. There are other proteins involved in double strand DNA repair and it is currently not known whether their expression would also be modulated by TH. Our findings further suggest that TH is able to protect the DNA integrity against the tamoxifen metabolite, OHT, in non-cancerous cells and thereby preventing initiation of a chain of reactions that transform these into cancer cells. This differs from the action of the phytoestrogen, genistein, which acts as an antioxidant that protected both normal and cancer cells against the genotoxic potential of tamoxifen [9]. Similarly, TH was shown to protect keratinocytes from DNA damage induced by ultraviolet radiation and the reduced pyrimidine dimer formation could be the result of enhanced repair [35]. Tamoxifen is capable of generating ROS *in vitro*[36] that would mediate the OHT-induced DNA damage observed. The protective effect of TH against this DNA damage in the non-cancerous cells could therefore be due to the ability of antioxidant compounds present in the honey to quench the ROS generated. This is supported by the study of Beretta et al. [37] that showed honey antioxidants protected cells against free radical scavenging action and boost intracellular GSH generation.

## Conclusions

The current study shows that TH enhances OHT-induced cytotoxicity and DNA damage in breast cancer cells while affording protection to the non-cancerous cells. This involves upregulation of double strand DNA repair enzymes that may thus increase the efficiency of DNA repair mechanism in these cells, and thereby preserving the cellular DNA integrity. The potential of TH to arrest breast cancer growth while protecting normal breast epithelium should be further explored *in vivo*. At least the use of TH may be able to reduce the toxic effects of a chemotherapeutic agent that could improve the quality of life for breast cancer patients.

## Abbreviations

TH: *Tualang* honey; OHT: 4-hydroxytamoxifen; ER: Estrogen receptor.

## Competing interests

The authors declare that they have no competing interests.

## Authors’ contributions

NF Ismail carried out the laboratory work, analysed the data and was involved in drafting the manuscript. NS Yaacob conceived and designed the study, interpreted the data and prepared the manuscript. Both authors read and approved the final manuscript.

## Pre-publication history

The pre-publication history for this paper can be accessed here:

http://www.biomedcentral.com/1472-6882/14/106/prepub
